# Effects of Concurrent, Within-Session, Aerobic and Resistance Exercise Training on Functional Capacity and Muscle Performance in Elderly Male Patients with Chronic Heart Failure

**DOI:** 10.3390/jcm12030750

**Published:** 2023-01-17

**Authors:** Maurizio Volterrani, Giuseppe Caminiti, Marco Alfonso Perrone, Anna Cerrito, Alessio Franchini, Vincenzo Manzi, Ferdinando Iellamo

**Affiliations:** 1 Department of Rehabilitation Cardiology, IRCCS San Raffaele Pisana, 00163 Rome, Italy; 2 Department of Clinical Science and Translational Medicine, University of Rome Tor Vergata, 00133 Rome, Italy; 3 Department of Humanities, Università Telematica Pegaso, 80132 Naples, Italy

**Keywords:** chronic heart failure, concurrent training, aerobic exercise, resistance exercise

## Abstract

Background. The best format of exercise training (ET) in the setting of cardiac rehabilitation in patients with chronic heart failure (CHF) is still to be defined. Current guidelines recommend aerobic exercises, such as running and cycling, including some sessions per week of resistance exercise. Aim. The aim of this study was to address the effectiveness of a concurrent exercise training program utilizing a circuit of sequential endurance and resistance exercises on functional capacity and muscular strength in patients with CHF. Methods. Ninety-five consecutive male patients (age 63.1 ± 6 years) with CHF (EF < 40%) in NYHA functional class II/III, were randomly assigned on 1:1 basis to a 12-week aerobic continuous training (AT) or concurrent CT), aerobic + resistance, training (CT), three times a week, with each session lasting 80 min. We used high quality, specifically designed ergometers, connected with each other and governed by a central console, and managed by a single physiotherapist. Before and after training all patients performed a symptoms-limited exercise test on a treadmill and a 6-min walking test (6MWT). Patients in the CT group also performed resistance exercises of upper and lower body. Results. The 6MWT and exercise duration at ergometric test increased significantly in both AT and CT groups, with the increase being greater in CT group (*p* < 0.001; ES = 0.13; *p* < 0.01; ES = 0.07). Muscular strength increased significantly in the CT group, particularly in the lower body muscular districts (*p* < 0.001). Quality of life improved in both groups, with a significantly greater improvement in the CT group (*p* < 0.05). No side effects leading to discontinuation of training were observed. Conclusions. These findings indicate that concurrent, within-session training results in larger improvements in functional capacity, in addition to muscle performance, in patients with CHF, in comparison to single-mode aerobic training.

## 1. Introduction

Exercise training (ET) is a well-established core component of cardiac rehabilitation in patients suffering from cardiovascular disease, including those with chronic heart failure (CHF) [1]. However, controversy still exists as to which ET program is more effective in CHF. To date, ET consisted substantially in endurance and aerobic exercises, e.g., running and cycling, with the aim of improving, among a number of beneficial effects, functional capacity and exercise tolerance, while reducing symptoms and improving quality of life. Current guidelines also recommend to include some sessions per week of resistance exercise, e.g., leg extension and bench press, with the aim of also improving muscle strength and power, in this frail and often aged patient’s population [1]. Indeed, combining endurance and resistance (i.e., concurrent training, CT) could be a promising way to optimize training. Combined training compared with single-mode endurance training may produce larger performance improvements in time trials in runners and cyclists [2]. In addition, when elite cyclists combined cycling and lower limb progressive resistance training, combined training improved mean power output during a 45 min cycle ergometer test more than did endurance exercise alone [3].

However, endurance and resistance exercises could also interfere with each other [4] and produce inferior gains in muscular strength compared with resistance exercise alone, giving origin to the so-called “interference effect” [5,6]. Interference occurs when strength and endurance stimuli both target peripheral (i.e., muscular) adaptations (e.g., hypertrophy vs. muscle capillarization) [4] and a meta-analysis confirmed the occurrence of the “interference effect” [6]. That is, strength training alone compared to combined training produced larger improvements in muscle strength and power. On the other hand, the opposite negative effect of the muscle interference, that is, a reduced endurance capacity with combined endurance and resistance exercises vs. endurance exercise alone, could also occur.

Despite the well-established effectiveness and safety of resistance exercises, specific recommendation as to the modality of their execution along with endurance exercise within cardiac rehabilitation programs are still lacking. Current indications include a set of exercise involving different muscle groups of upper and lower body (e.g., chest press, leg extension, etc.) at a relative intensity of 40–60% or 60–80% of one repetition maximum (1RM) to be performed twice/three times a week [1]. Main problems are represented by the relative low quality of the equipment used to perform resistance exercise, utilizing weight machines system and pulleys of dubious quality, often not allowing an accurate quantification of the maximal voluntary contraction.

Resistance exercises in cardiac rehabilitation programs are usually performed on alternative days with aerobic ones during the training weeks [1]. Whether accomplishing the two types of exercise within the same daily sessions would improve muscle strength and/or functional capacity more than when both types of exercise are performed on alternative days, to our knowledge, has not been investigated. Previous studies [7] comparing CT versus AT did not employ within-session training of both types of exercise, and reported conflicting results as to the effects of CT vs. AT on functional capacity [7]. 

The aim of this study was to address the effectiveness of a concurrent exercise training program utilizing a circuit of sequential endurance and resistance exercises, involving the main body muscle groups, on functional capacity and muscular strength in patients with CHF. For this purpose, we used high quality, specifically designed ergometers for the different muscular districts, connected with each other and governed by a central kiosk, able to manage personalized endurance and resistance exercise training programs within the same session, and guided by a single physiotherapist (see methods). This would have a greater impact on functional capacity and in patients with CHF.

The hypothesis tested was that concurrent training is more effective than endurance training alone in improving both functional capacity and muscle strength and power.

## 2. Materials and Methods

### 2.1. Patients and Study Design

We enrolled 95 consecutive male patients (age 63.1 ± 6 years) with post-infarction heart failure referred to our center for a cardiac rehabilitation program. Subject eligibility was determined at the initial screening visit. Patients were included in the study if they had left ventricular ejection fraction (LVEF) < 40% at echocardiographic examination, heart failure with functional New York Heart Association (NYHA) class II or III, clinical stability without hospital admission for HF in the previous 3 months, sinus rhythm and were on optimal medical treatment for at least three months. Patients were excluded if they had unstable angina or recent acute myocardial infarction (less than 6 months), frequent atrial or ventricular premature beats, conduction defects, pacemaker, uncontrolled hypertension, history of severe kidney diseases (GFR < 30 mL/min), significant pulmonary disease (FEV1 < 50%), severe lower extremities vascular or other diseases which could prevent a symptom limited exercise test, coexisting valvular disease and insulin-dependent diabetes. Medications were not altered throughout the study.

Patients were randomly assigned on 1:1 basis to a 12-week AT or concurrent, aerobic + resistance training (CT). All subjects performed a symptoms-limited exercise test on a treadmill (Mortara Instrument, Casalecchio Di Reno, Italy) using a standard Bruce protocol. The test was started at a speed of 1.7 mph and inclination of 10%. Speed and inclination were gradually increased every 3 min. Continuous 12-leads electrocardiographic (ECG) monitoring was performed throughout the test. Blood pressure (BP) were measured at rest, at each stage of the exercise phase and during the recovery by using a manual sphygmomanometer. In both groups, training program consisted in three sessions per week, and each session lasted 80 min.

### 2.2. Echocardiographic Assessment

Before and after training, all patients underwent a complete two-dimensional, M-mode, and Doppler echocardiogram using an Acuson Sequoia device and a 2.5–4.25 MHz wide-angle phase array transducer. Left ventricular volumes were measured from the apical four- and two-chamber views. Left ventricular ejection fraction (LVEF) was calculated using the Simpson rule algorithm.

### 2.3. Exercise Training Protocols

Aerobic Training: Each exercise session included 10 min of warm-up, 10 min of cool-down and 40 min of aerobic exercise on a treadmill or bicycle. The exercise intensity was established by means of RPE [8]. Patients were instructed and familiarized with the use of the Borg 6–20-points scale before the beginning of the training. At the first session, the treadmill was set to a level of velocity and slope correspondent to an RPE of 13–14 (somewhat hard) for each patient, according to the individual results at the baseline exercise test. The instruction from the physiotherapist was to exercise at RPE 13–14 during the whole study and patients were free to change the treadmill setup during subsequent sessions in order to maintain the same level of effort. The RPE method was chosen as to permit updating exercise prescriptions as fitness levels changed.

From the average HR data recorded during each exercise session, the corresponding VO_2_ and energy expenditure was estimated using the individual HR–VO_2_ regression equations obtained in laboratory [9].

Concurrent training: Patients performed aerobic exercises before resistance exercises in each session. In order to balance the total amount of exercise between the two groups, exercise sessions in the CT group were planned as follows: 20 min of aerobic training on treadmill; and 20 min of resistance training consisting in the following exercises: leg press and extension, shoulder press, chest press, low row and vertical traction (Technogym Wellness System, Technogym, Cesena, Italy). As for the endurance group, the intensity of the aerobic component of training was established by means of the RPE method with an intensity target of 13–14 during the whole study. 

For resistance exercises, each muscle group involved in the training was preliminary tested for 1 RM. 1 RM had been determined, for each muscle group, as the highest force developed by the patients in three previous 5-s maximal contraction trials. Particular attention was paid to avoid the contraction of muscle groups other than those specifically involved in the exercise (that is, accessory muscle recruitment).

Ergometers employed in this study have the capability of detecting (and recording) directly the 1 RM. This permits a greater accuracy in the determination of RM and considerable time saving in comparison to the use of repetitions of successively higher weights for determination of 1 RM.

Patients performed 10 repetitions per set, 2 sets for each exercise at 60% of 1RM. Patients in the CT group also performed 10 min of warm-up and 10 min of cool-down. Each participant was assigned a key that stored their individualized workout prescription that was programmed under the guide of physical therapists and supervised by a cardiologist. Participants checked in using their key at a computer kiosk, which recorded attendance automatically. After checking in, the participant inserted their key into each exercise machine, and their workout prescription was loaded onto the machine [e.g., treadmill speed and grade are automatically controlled and adjusted following the stored exercise program in their key and each participant’s heart rate (HR) during exercise]. After each exercise workout, performance data (e.g., HR, duration, sets, repetitions, strength) were stored in the key. Participants checked out with their key, and their exercise data were transmitted back to a local database. 

The sequence of exercise was as follows: Treadmill or Bike, Chest Press, Low Row, Vertical Traction, Leg Extension, Shoulder Press, Leg Press.

During all exercises, heart rate was recorded using short-range telemetry (Polar Team System, Polar Electro Oy, Kempele, Finland). 

Quality of Life (QOL) was also assessed through a vertical visual analog scale at baseline and at the end of 12 weeks exercise programs. The visual analog scale was a 10-cm line with a mark at each centimetre [10].

All patients gave written informed consent to participate in the study, which was approved by the Ethics Committee of IRCCS San Raffaele Pisana (protocol code 23/2019). The study was conducted in accordance with the Declaration of Helsinki.

### 2.4. Statistical Analysis

Based on the results of previous studies [7], we estimated that a sample size of 42 subjects per group had 80% power to detect a between-group difference on the increase of maximal exercise duration at ergometric test of 10–8%, with a standard deviation of 3%, using a two-sided significance level of 0.05. We estimated the drop-out rate to be 15% leading to an overall sample size of 95 patients. Data are presented as mean ± SD or percentage, where appropriate. Preliminary assumption testing was conducted to check for normality, linearity, univariate and multivariate outliers, homogeneity of variance-covariance matrices, and multicollinearity. A mixed between-within subject analysis of variance was conducted to assess the impact of the two different training programs (AT, CT) on participants’ physical and physiological parameters across two time periods (pre-training and post-training). Pretraining-to-posttraining changes in muscular strength variables within CT group were examined with paired *t*-tests. Unpaired *t*-tests were performed to examine between AT and CT groups difference in the energy expenditure. Practical significance was assessed by calculating the Cohen’s *d* effect size [11] (>0.8, between 0.8 and 0.5, between 0.5 and 0.2, and <0.2 were considered as large, moderate, small, and trivial) and partial eta squared (η_p_^2^) (<0.01—very small; from >0.01 to <0.06—small; >0.06 to <0.14 –moderate; and >0.14—large). A significance level of *p* < 0.05 was selected. All analyses were performed using a commercially available statistical package (SPSS for Windows 25.0, Chicago, III).

## 3. Results

Baseline characteristics of the two groups are summarized in Table 1. No significant differences were detected between the two groups in any variable.

**Figure 1:**
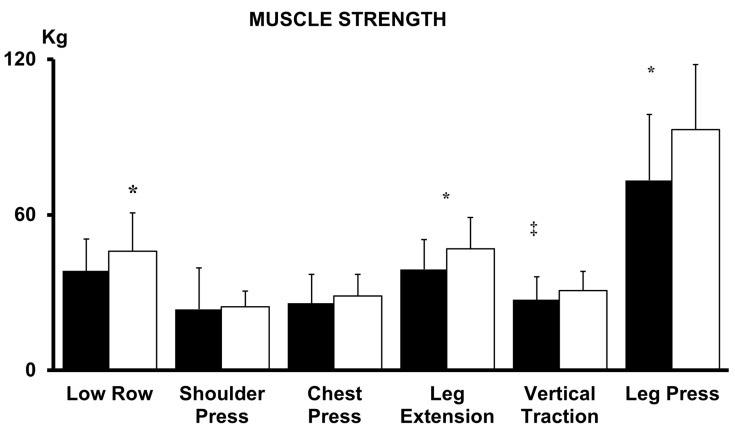
Muscle strength at baseline (black bars) and after 12 weeks of training (white bars) in CT group. (^‡^ *p* < 0.01, * *p* < 0.001 versus baseline, Low Row, ES = 0.55; Shoulder Press, ES = 0.07; Chest Press, ES = 0.27; Leg Extension, ES = 0.66; Vertical Traction, ES = 0.43; Leg Press ES = 0.76).

The 6MWT and exercise duration at maximal ergometric test increased significantly in both AT and CT groups (*p* < 0.001; ES = 0.78 large; *p* < 0.01; ES = 0.62 large) but the increase was greater in magnitude in CT group (*p* < 0.001; ES = 0.13 moderate; *p* < 0.01; ES = 0.07) (Table 2). Resting heart rate (HR) systolic (S) and diastolic (D) blood pressure (BP) were reduced significantly by both AT and CT training (*p* < 0.01; ES = 0.08 moderate; *p* < 0.01; ES = 0.07 moderate; *p* < 0.001; ES = 0.11 moderate), without significant between-groups differences (Table 2). EF did not change significantly with training in both groups (*p* > 0.05, Table 3). As expected, muscular strength increased significantly with the biocircuit training, particularly in the lower body muscular districts, whereas it was less significant or not significant in the upper body muscles (Figure 1). Mean estimated oxygen uptake in the AT patients during each session, based on the individual’s relationship between heart rate and oxygen uptake obtained from the treadmill test, was 12.06 ± 0.82 mL·kg^−1^·min^−1^ (68.0 ± 3.1% of maximal oxygen uptake), which corresponded to average session energy expenditure of 205 ± 27 Kcal. The average energy expenditure per training session for patients in the CT group was of 197 ± 32 Kcal (aerobic training 89 ± 23 resistance training 99 ± 18 Kcal). Energy expenditure did not differ significantly between AT and CT groups (*p* > 0.05; ES = 0.27). In our study there was a high compliance: overall only 8% of patients were lost during the follow-up. The percentage of attendance to training sessions (number of sessions attended/prescribed) was slightly higher in the CT compared to AT group (97.3% and 91.6% respectively; *p* = 0.08). QOL showed an improvement in both training groups, with a greater improvement in the CT (from 4.2 ± 0.7 to 6.0 ± 1.1) compared to the AT group (from 4.1 ± 0.5 to 5.3 ± 0.9) (between-groups difference *p* < 0.01). No side effects leading to discontinuation of training were observed throughout the study.

## 4. Discussion

The main finding of this study is that concurrent training resulted in larger improvements in functional capacity and muscle performance in comparison to single-mode aerobic training in patients with CHF. To our knowledge, this is the first study to compare AT vs. concurrent (i.e., within-session) AT plus resistance exercise training program in CHF patients. Previous studies investigated the effects of combined, not concurrent, AT plus resistance vs. AT training alone provided conflicting results [12,13,14]. Beckers et al. [12] found a greater increase in sub-maximal exercise in the CT compared to AT, while gain in peak exercise capacity were similar between the two groups. In the study of Mandic et al. [14] VO2 peak increased significantly only in the AT group. In our study, we observed a greater increase in exercise capacity in the CT group. Differences in study design (e.g., exercise protocols, duration of the studies, sample size) together with a low-quality machines might be responsible for the different findings. In the study of Beckers et al. [12], the portion of the exercise sessions dedicated to resistance exercises in the CT group was greater at the beginning of the training protocol and afterwards was gradually shortened in favour of a longer aerobic portion. Conversely, in our study the time spent respectively on resistance and aerobic exercises in each session was constant during the entire training period. It should be also mentioned that the study of Beckers et al. [12] was longer (6 months) than the other ones, included the present investigation. Our results would indicate that benefits related to concurrent exercise training are evident already after few weeks.

Findings of our study confirm and extend to concurrent training modality, the recommendation to include resistance exercises in the training of CHF patients [1], even though direct evidence for this was still scant. Generally, resistance exercise is recommended as an adjunct to aerobic exercise to improve muscle performance inasmuch as resistance exercise alone does not improve functional capacity in patients with CHF. The limited available data offers a little insight into the optimal prescriptive principles for exercise training. Our results suggest that combining resistance and aerobic exercise, i.e., concurrent training, within the same training session enhances functional capacity (in addition to muscles strength), as indicated by the increase in 6MWT, more than did aerobic exercise alone, likely through an increase in exercise economy. Additionally, the duration of exercise at the maximal exercise test to exhaustion was greater in the CT than AT group, although not significantly, indicating a delayed fatigue. This finding would confirm in patients with CHF the results of several previous studies conducted in athletes [2,15,16,17,18,19].

EF and cardiac chambers dimensions did not change significantly with both AT and CT, in line with current findings [12,14], indirectly suggesting that the beneficial effects of exercise training on functional capacity in patients with CHF would be mainly due to peripheral mechanisms and adaptations [20,21,22,23], although not invariably [24,25]. Similarly, the blood pressure lowering effect was not significantly different between the two groups.

As far as the muscular effects is concerned, we observed an increase in muscles strength in all muscular districts investigated, which was particularly significant for the lower body (e.g., Leg Extension and Leg Press) whereas it was less significant or not significant for the trunk muscles (Figure 1). It thus appears that the exercise-induced increase in muscular strength occurs mostly in weight-bearing muscles, which are those more relevant to the effort intolerance and symptoms development of the CHF syndrome.

The increase in muscle performance observed after a training program including resistance exercises would have been to be intuitively expected, yet it was to be demonstrated, inasmuch as it was imbricated with aerobic exercises, within the biocircuit training, and it has been repeatedly reported that combining aerobic and resistance exercise could reduce the effects on muscle performance induced by resistance exercise, the so-called “Muscle Interference” phenomenon [6,26,27,28,29,30].

Studies in athletes consistently reported that after CT intervention muscle hypertrophy, strength and power adaptations were mostly attenuated, compared with those after isolated strength training [26,27,28,29,30] even though others did not found evidence that resistance training adaptations are suppressed, with CT [31,32]. Thus, it seems that CT does not negatively affect the endurance training adaptations [30,33]. However, it has been recently suggested that longitudinal CT programs may impair the endurance training adaptations in well-trained runners [33].

Our results contradict the “Muscle Interference” hypothesis, since CT did not oppose an increase in muscle strength, despite it included aerobic exercises, but also resulted in an increase in functional capacity in patients with clinically stable CHF. Hence, we found at worst no interfering but a potentiating effect of CT compared with AT alone in CHF patients.

Objective morphological and functional abnormalities, relatively independent of reduced blood flow, are present in the muscle of CHF patients [33] and endurance exercise training has been shown to improve muscle structure and function, in addition to VO_2_ in CHF [33,34,35,36,37,38,39,40].

It is arguable that molecular, metabolic and functional alterations occurring in the muscles of CHF patients, make them more suitable to respond positively to combined exercise training, although the mechanism(s) responsible for these adaptations cannot be elucidated by the present study and warrant further investigations. Indeed, there is suggestion that in previously untrained/recreational individuals there is a greater increase in all molecular pathways controlling both myofibrillar and mitochondrial protein synthesis after a CT, leading to significant adaptations with both regimens, compared to a single bout of either resistance or endurance exercise [41,42,43,44,45,46]. We purposely did not include in this study a patients’ group performing resistance training alone, since it is well established that resistance exercise alone does not affect functional capacity, the variable of major relevance in CHF. Indeed, utilization of strength training exercises alone is rarely applied in the setting of cardiac rehabilitation programs. Within this framework, we also choose that our patients had to perform endurance exercises before resistance exercises, according to the priority training principle that exercises aiming to improve the most important determinant parameters of performance should be performed first [47], even though we did not provide direct evidence for this.

Strength of the present study in comparison to previous ones is in that both aerobic and resistance exercises were performed with the aid of a specifically designed, commercially available, computer-driven circuit of high accuracy ergometers, capable of recording and storing individual muscle performance data. This approach also allows a considerable sparing of time and of physical therapists, being therefore cost effective.

Other strengths of the study were the control of training volume between CT and AT, so that any potentiating or interfering effect would be not due to differences in training volume between groups and the use of a HR monitoring device and therapist’s supervision in all training sessions, which allowed the verification of full adherence of patients to prescribed training protocols, not just attendance.

Limitations of the study. The main limitation of the present investigation is the relatively small sample size. As such, this investigation should be regarded as a “proof-of-concept” study. In addition, we cannot comment on clinical outcomes. The study was not designed for this purpose. However, the present study indicates that CT improves clinical measures that carry adverse effects and negative prognosis in CHF. We cannot comment on possible gender-related differences, since patients involved in the study were only males. Female patients attending exercise training programs are much fewer than males. This occurs all around the world because of a variety of reasons, as outlined in a recent editorial [48]. Since we did not expect to enrol a decent number of female patients, we decide to limit our intervention only to males. In this study we observed an improvement of muscle strength in the CT group that seems to exclude the muscle interference phenomenon. However given the actual design of our study muscle interference was not directly assessed since we did not perform a direct comparison between CT and isolated resistance training. Also, this trial lacks a control group of non-exercising patients. However, within the framework of the present investigation, this would be more a theoretical rather than an actual limitation. Indeed, it would be hard to hypothesize changes in muscle strength and functional capacity as those observed in our study over a three-month period without exercising or simply as a result of time. Anyway, the lack of a control non-exercising group was unavoidable in this study, because all the patients were expressly referred to an exercise-based cardiac rehabilitation program by their cardiologist or primary care physician, under reimbursement of the Italian Health Care system.

Lastly, we did not directly measure VO_2_ peak but assessed maximal exercise tolerance trough ergometric test duration and METs. Assessment of exercise capacity by means of effort tolerance tests such as the 6MWT is easy to perform, correlates with peak VO_2_, and has been shown to be useful in prognostic stratification [49,50,51,52,53,54,55].

Finally, as previously mentioned, the increase in muscle strength we observed after CT was likely related to changes in skeletal muscle ultrastructure and biochemistry, but we have no direct data about these changes.

## 5. Conclusions

In conclusion, the present study indicates that CT was superior to AT in improving functional capacity, in addition to muscles performance, in patients with CHF. This finding has practical implications because it indicates that exercise training within cardiac rehabilitation programs in older patients with CHF should include within session endurance and resistance exercises, in order to improve functional capacity and muscular performance. Future, larger studies are needed to confirm these results and, importantly, to inform us on clinical outcomes. The biocircuit modality with high-quality, computer-driven and -controlled ergometers could provide new insights on relevant physiological aspects of exercise training which could contribute to future guidelines on physical activity in cardiac patients.

## Figures and Tables

**Table 1:** Baseline characteristics of patients.

	Overall Population (n = 95)	Aerobic (n = 40)	Concurrent (n = 55)
Age, y	63.1 ± 6	62.6 ± 6	63.7 ± 2
BMI, Kg/m^2^	27.6	27.8 ± 2	27.2 ± 3
NYHA class II/III	2.6 ± 0.5	2.8 ± 1.1	2.4 ± 0.8
Resting HR, bpm	68.7 ± 13.4	66.9 ± 11.1	69.0 ± 17.2
Systolic BP, mmHg	123.3 ± 38.5	123.3 ± 38.5	123.3 ± 38.5
Diastolic BP, mmHg	83.5 ± 11.8	85.6 ± 16.3	82.0 ± 14.1
Previous CABG/PCI	43/68	19/30	24/38
**Comorbidities**			
Hypertension	72	30	42
Diabetes	31	12	19
COPD	16	6	10
Carotid artery disease	28	11	17
History of smoke/active smoker	71/9	30/5	41/4
**Echocardiography**			
LVDD, mm	58.1 ± 5	58.6 ± 7	57.3 ± 8
LVSD, mm	45.9 ± 4	45 ± 5	46 ± 5
Ejection fraction, %	42.0 ± 9	42.6 ± 9	41.8 ± 12
TAPSE, mm	21.3 ± 4	20.7 ± 7	22.0 ± 5
**Treatment**			
Beta-blockers	83	35	48
ACE-i/ARBs	76	32	44
Diuretics	47	20	27
Aldosteron-antagonists	67	28	39
Statins	71	30	41
Sacubitril/valsartan	12	4	8
Ivabradine	7	2	5

BMI, body mass index; CABG, Coronary artery bypass graft; PCI, percutaneous coronary intervention; LVDD, left ventricular diastolic diameter; LVSD, left ventricular systolic diameter; TAPSE, tricuspid annulus plane systolic excursion.

**Table 2:** Changes in hemodynamic and exercise tolerance parameters at baseline and after training.

	Aerobic Training (AT)		Concurrent Training (CT)		Group Post-Training Magnitude of Difference	
Exercise Tolerance	Baseline	After	Baseline	After	Effect Size	Interpretation
6MWT (m)	435.4 ± 49.4	498.3 ± 52.1 *	448.6 ± 42.7	558.4 ± 63.0 *^,†^	0.13	Moderate
Exercise duration at exercise test (s)	407.3 ± 49.4	441.9 ± 61.3 *	402.5 ± 48.2	479.0 ± 55.7 *^,‡^	0.07	Moderate
**Hemodynamic**						
HR (bpm)	66.9 ± 11.2	63.8 ± 16.2 *	69.0 ± 17.2	64.2 ± 16.3 *	0.002	Trivial
Peak HR (bpm)	128.3 ± 31.0	134.5 ± 33.0	127.9 ± 24.0	137.2 ± 37.3	NS	Trivial
SBP (mmHg)	123.3 ± 28.5	117.0 ± 26.1 *	122.7 ± 28.7	113.0 ± 21.3 *	0.003	Trivial
DBP (mmHg)	83.5 ± 11.8	79.5 ± 9.2 ^‡^	82.0 ± 14.1	79.0 ± 7.4 ^‡^	0.002	Trivial

6MWT¸ six Minute Walking Test; HR, heart rate; SBP, systolic blood pressure; DBP, diastolic blood pressure. * *p* < 0.001 vs. Baseline; ^†^ *p* < 0.001 vs. Aerobic training; ^‡^ *p* < 0.01 vs. Aerobic training; NS not significant.

**Table 3:** Changes in echocardiographic parameters at baseline and after training.

	Aerobic Training (AT)		Concurrent Training (CT)	
Echocardiography	Baseline	After	Baseline	After
LVEDV (mL)	164.3 ± 37.3	161.7 ± 48.5	162.6 ± 44.2	160 ± 39.7
LVESV (mL)	74.1 ± 18.5	73.2 ± 13.9	73.9 ± 19.4	71.5 ± 17.3
LVEF (%)	42.6 ± 9.2	43.0 ± 11.4	41.8 ± 12.3	42.7 ± 10.1
LV GLS (%)	−10.8 ± 2.1	−11.6 ± 2.8	−11.0 ± 1.9	−11.9 ± 2.2
E/E’	7.5 ± 1.7	8.1 ± 2.4	7.6 ± 1.1	9.2 ± 1.6
E (cm/s)	69.0 ± 21.3	64.3 ± 24.1	69.5 ± 18.7	64.8 ± 15.6
A (cm/s)	68.5 ± 16.8	68.3 ± 18.3	70.8 ± 19.5	71.1 ± 16.2
E’(cm/s)	9.1 ± 1.5	8.7 ± 2.2	9.5 ± 1.9	8.5 ± 1.4
TAPSE (mm)	20.7 ± 7.0	20.4 ± 4.4	21.0 ± 5.8	21.5 ± 6.3

LVEDV, left ventricular end diastolic volume; left ventricular end systolic volume; LVEF, left ventricular ejection fraction; LV GLS, left ventricular global longitudinal strain; TAPSE, Tricuspid Annular Plane Systolic Excursion. Differences are not statistically significant.

## Data Availability

The data presented in this study are available on request from the corresponding author.
